# The Association of Caregivers’ Socio-Economic Conditions with Family Caregiving Norms: Evidence from China

**DOI:** 10.3390/bs13050362

**Published:** 2023-04-26

**Authors:** Yu Kuramoto, Honoka Nabeshima, Mostafa Saidur Rahim Khan, Yoshihiko Kadoya

**Affiliations:** School of Economics, Hiroshima University, 1-2-1 Kagamiyama, Higashihiroshima 7398525, Japan; b206431@hiroshima-u.ac.jp (Y.K.); b202314@hiroshima-u.ac.jp (H.N.); ykadoya@hiroshima-u.ac.jp (Y.K.)

**Keywords:** eldercare in China, family caregiving norms, caregivers’ socio-economic conditions, aging population

## Abstract

Similar to her neighboring country, Japan, China faces significant difficulties in providing long-term care to the elderly. Female household members who traditionally provided necessary caregiving are no longer available as much as in the past due to the demographic and socioeconomic changes over the past few decades. Against this backdrop, we investigated how socioeconomic factors affect the perception of family caregiving norms in China, using an international comparative household dataset that allowed us to compare China with Japan, the latter being extensively investigated. We used ordered probit regression to estimate the model equation. Our results show that rural residency, household assets, and government dependency are positively associated with the perception of care. A notable difference from the Japanese results is that rural residents have a rather positive perception of family caregiving norms. Furthermore, urban–rural subsample analyses revealed that women in rural areas perceive caregiving negatively.

## 1. Introduction

A recent study in Japan found that age, education, full-time employment, marriage, and the number of children of potential caregivers are negatively associated with the attitude of family caregiving [1]. The results imply that changes in demographic and socioeconomic conditions, women’s participation in the formal labor market, and the influence of the global diffusion process act as agents of change to traditional family caregiving norms. This study [1] motivated us to investigate the perception of traditional caregiving norms in light of the changing demographic and socio-economic conditions in China. China, which is a neighboring country of Japan, shares commonalities in family caregiving values, practices, and challenges. For example, both Japan and China follow perceptions of family caregiving that are deeply rooted in tradition [2], where the influence of filial piety requires children to take care of elderly family members and rely less on institutional care [3,4]. However, the way socioeconomic and demographic shifts have impacted China has some unique characteristics, and therefore, its impact on societal values must be studied separately and rigorously [5,6,7,8,9]. Furthermore, the rural–urban dimension is particularly important to study in China due to the difference in socio-economic conditions, cultural coherence, and institutional structures observed during the last couple of decades [10]. Therefore, a comprehensive study of the perception of caregiving in China will allow people to understand the contrasting influence of traditional filial piety on caregiving and the actual perception of family caregiving under changing demographic and socio-economic conditions.

Family caregiving is an important source of elderly care not only due to the influence of culture but also due to the lack of institutional care and its affordability at the mass level in China [11]. Not very surprisingly, China depends heavily on family caregivers to provide daily and instrumental elderly care [12]. Moreover, eldercare recipients primarily prefer family members as their caregivers due to cultural and comfort issues [13]. Expectations of both caregivers and care recipients are largely dominated by their filial relationship [3,4]. However, the traditional supply of family care is facing increasing challenges due to the changing family structure; increasing employment of women, who are the main providers of family care; increasing socio-economic status; and reducing the number of eligible family members [14,15,16,17,18]. Female household members, who traditionally provided the necessary family care [19], are no longer available as much as in the past due to the demographic and socio-economic changes over the past few decades [20,21,22]. Socio-economic changes have also influenced caregivers’ physical and psychological burdens associated with caregiving responsibilities, which eventually have impacted caregivers’ perception of caregiving norms [23,24,25,26].

The existing literature focuses mainly on the influence of tradition and culture on family caregiving [27,28,29], the provision of eldercare by institutional providers and its barriers [30,31,32,33], and stress and burden associated with family caregiving [34,35,36]. However, there is a lack of study on how the changes in socio-economic conditions are associated with caregivers’ attitudes towards family caregiving norms. The massive socio-economic development over the last couple of decades, combined with a rapidly aging population and the implementation of the one-child policy, has placed China in a critical stage where traditional caregiving norms have been severely impacted, which could have severe consequences for elderly care in the future. Thus, the reasons behind developing negative attitudes towards family caregiving norms must be investigated and understood from the viewpoint of caregivers’ needs and burdens.

This study examines the association between various demographic and socio-economic factors and the Chinese perception of family caregiving norms with a particular focus on differentiating between rural and urban areas. This study contributes to the literature in at least three ways. First, it quantitatively demonstrates how various demographic and socioeconomic conditions are associated with the perceptions of family caregiving norms in China. Second, using the same series of datasets, this study produced outputs comparable to those of Japan, which shares similar issues with China. Third, this study uniquely covers rural–urban differences using nationwide survey data.

## 2. Data and Methods

### 2.1. Data

This study used data from the Osaka University’s Preference Parameters Study, an international comparative household survey. The panel survey distributed a nearly identical questionnaire in four countries, including China and Japan. We used data from the 2013 wave, which provided necessary information for comparison. The Chinese survey was conducted through face-to-face interviews with individuals and households in six major cities—Beijing, Shanghai, Guangzhou, Chengdu, Wuhan, and Shenyang—and the surrounding rural areas from 17 January 2013 to 1 February 2013. The target respondents were adults aged 20–69 years old. The survey used multistage sampling and allocation methods. Specifically, the first study set predicted the number of respondents based on the target population in each district. Subsequently, an area in each district was selected randomly. Finally, the potential participants were selected from families using the Kish grid method [37]. As the focus was on major Chinese cities, the data do not represent the entire nation, but they include significant parts of the country. There were two separate datasets—one each for rural and urban areas. We appended the two datasets to perform relevant analysis. We excluded 58 respondents with missing data on socioeconomic and demographic variables. The final sample size was 1260, accounting for approximately 96% of valid respondents out of a total of 1318 respondents.

### 2.2. Variables

The dependent variable was perception of family caregiving norms. To quantify this, we used the following question: “Child(ren) should take care of their parents when they require long-term care”. Respondents had the options to answer this question on a scale of 1 to 5, with 1 being completely disagree and 5 being completely agree.

The explanatory variables of this study were grouped into five categories. The first group includes demographic characteristics of the respondents, such as sex, age, level of education level, religious level, employment status, marital status, and some important interaction terms such as sex with age and sex with full-time employment. The second group includes variables related to potential caregivers, such as the number of siblings, the number of sons, and the number of daughters. The third group includes variables related to respondents’ financial conditions, such as the logs of household annual income and household financial assets. The fourth group includes the financial planning for old age, such as the proportion of living expenses that the respondents will be able to cover with social security income after retirement, the perception of the government’s role in taking care of financially dependent people, having savings for living expenses, and having savings for long-term care. The fifth group includes parental-related variables, such as whether the respondents received an inheritance or monetary transfers in excess of CNY 100,000 from their parents, the level of education of the parents, whether one parent requires care, and whether both parents require care. Most of our explanatory variables are comparable to those of Fukuda [1] and Khan et al. [38]. Table 1 presents the definitions of all the variables.

### 2.3. Descriptive Statistics

Descriptive statistics of the socioeconomic and demographic characteristics of the respondents are presented in Table 2. The statistics revealed that respondents were mostly in favor of family caregiving norms (mean 4.11, SD = 74), indicating strong filial piety norms embedded in the Chinese culture. Approximately 35.6% of the sample lived in rural areas, and 50% of the respondents were female, with an average age of 44 years. On average, the respondents had been educated for 10.3 years. In addition, 2.2% of the respondents were unemployed, and 81% were married. The respondents had on average two siblings and fewer than one son or daughter. Furthermore, the average household income and assets were more than CNY 1,600,000 and CNY 8,000,000, respectively. Respondents expected that after they retired, they could cover 23.8% of their living expenses using their social security income. Moreover, 77.4% of respondents believed that the government should take care of those who cannot take care of themselves financially. With regard to savings, 35.7% had saved for the living expenses during old age, and 24.6% had saved for nursing and other expenses in long-term care. Additionally, 7.8% of respondents had received an inheritance worth more than CNY 100,000 from their parents. The average number of years of education for the respondents’ parents was 6.5 years. Finally, 20.6% of the respondents reported that one parent required long-term care, whereas 11% stated that both parents needed caregiving.

We observed whether perception of family caregiving norms varies with individuals’ socioeconomic and demographic characteristics, as presented in Table 3 and Table 4 below.

Caregiving perceptions according to age group are presented in Table 3. Age is known to be the best indicator of a person’s behavior in various social contexts. However, the ANOVA test in our study showed that age was not significantly associated with the caregiving perception in China. This result indicates that age, sometimes considered the only predictor, might not explain certain relationships depending on the stance of the subject area.

There was a significant disparity between rural and urban areas in the perception of family caregiving norms, as presented in Table 4. People living in rural areas have a more positive perception of caregiving norms than those in urban areas do. The noteworthy difference can be attributed to fewer healthcare services vis-à-vis a greater need for care in rural areas, the higher influence of filial piety norms among the residents of rural areas than among those in urban areas, and other sociocultural factors that shape the household structure in both. By contrast, other categories such as gender, marital status, and employment status did not affect respondents’ attitudes toward long-term care. This implies that these variables might not be strong predictors of certain correlations and could be combined with other demographic and socioeconomic variables to demonstrate a joint effect.

### 2.4. Methods

We investigated the association between respondents’ perceptions of family caregiving norms and various socioeconomic and demographic attributes using Equation (1). As the main dependent variable is ordinal, we conducted an ordered probit regression to estimate the equation. Ordered probit model is a generalization of probit model and applicable where the dependent variable has more than two outcomes of ordinal in nature. The ordered probit model appropriately fits ordered data, preserving the ordering of response options when making no assumption of the interval distance between options [39].

(1) $$Y_{i}=f(,X_{i},\;\varepsilon_{i})$$

where $Y_{i}$ is the perception of the family caregiving norms of the ith respondent, X is the vector of respondents’ socio-economic and demographic characteristics and some interaction terms, and ε is the error term.

With several predictor variables in the model, the probability that our model could suffer from multicollinearity was high. This is because the model comprises variables that might reinforce one another; for example, higher years of education may lead to higher household assets or income or vice versa. We conducted correlation and multicollinearity tests (available upon request) to ensure that this inter-correlation did not persist in our model. The correlation matrix shows a weak relationship between the relative movements of the two variables across all the models (substantially lower than 0.70). Furthermore, the variance inflation factor values of all the explanatory variables were less than 10, indicating the absence of multicollinearity in all models.

Five models were created for Equation (1), with each model adding distinct control variables, which are represented in models 2, 3, 4, 5, and 6 below.

(2) $$Perception\;of\;family\;caregiving\;norm_{i}=\beta_{0\;}+\beta_{1}rural_{i}\;+\beta_{2}female_{i}+\beta_{3}age_{i}+\beta_{4}age\_sq_{i}+\beta_{5}years\_of\_education_{i}+\;\beta_{6}unemployed_{i}+\beta_{7}married_{i}+\beta_{8}cross\_fem\&agesquared_{i}+\;\varepsilon_{i}$$

(3) $$Perception\;of\;family\;caregiving\;norm_{i}=\beta_{0}\;+\beta_{1}rural_{i}\;+\beta_{2}female_{i}+\beta_{3}age_{i}+\beta_{4}age\_sq_{i}+\;\beta_{5}years\_of\_education_{i}\;+\beta_{6}unemployed_{i}\;+\beta_{7}married_{i}\;+\;\beta_{8}cross\_fem\&agesquared_{i}+\beta_{9}nb\_siblings_{i}+\beta_{10}nb\_sons_{i}+\;\beta_{11}nb\_daughters_{i}+\varepsilon_{i}$$

(4) $$Perception\;of\;family\;caregiving\;norm_{i}=\beta_{0\;}+\beta_{1}rural_{i}\;+\beta_{2}female_{i}+\beta_{3}age_{i}+\beta_{4}age\_sq_{i}+\beta_{5}years\_of\_education_{i}\;+\beta_{6}unemployed_{i}+\beta_{7}married_{i}+\beta_{8}cross\_fem\&agesquared_{i}+\;\beta_{9}nb\_siblings_{i}+\beta_{10}nb\_sons_{i}+\beta_{11}nb\_daughters_{i}\;+\;\beta_{12}\log of\;household\;income_{i}+\;\beta_{13}\log of\;household\;asset_{i}\;+\varepsilon_{i}$$

(5) $$Perception\;of\;family\;caregiving\;norm_{i}=\beta_{0}\;+\beta_{1}rural_{i}\;+\beta_{2}female_{i}+\beta_{3}age_{i}+\beta_{4}age\_sq_{i}+\beta_{5}years\_of\_education_{i}+\;\beta_{6}unemployed_{i}+\beta_{7}married_{i}\;+\;\beta_{8}cross\_fem\&agesquared_{i}+\;\beta_{11}nb\_siblings_{i}+\beta_{12}nb\_sons_{i}+\beta_{13}nb\_daughters_{i}+\;\;\beta_{12}\log of\;household\;income_{i}+\beta_{13}\log of\;household\;asset_{i}\;+\;\beta_{14}social\_security_{i}+\beta_{15}gvt\_responsibility_{i}+\;\beta_{16}save\_retire_{i}+\beta_{17}save\_ltc_{i}+\varepsilon_{i}\;$$

(6) $$Perception\;of\;family\;caregiving\;norm_{i}=\beta_{0}+\beta_{1}rural_{i}\;+\beta_{2}female_{i}+\beta_{3}age_{i}+\beta_{4}age\_sq_{i}+\beta_{5}years\_of\_education_{i}\;+\beta_{6}unemployed_{i}+\beta_{7}married_{i\;}+\;\beta_{8}cross\_fem\&agesquared_{i}+\;\beta_{9}nb\_siblings_{i}+\beta_{10}nb\_sons_{i}+\beta_{11}nb\_daughters_{i}\;+\;\beta_{12}\log of\;household\;income_{i}+\beta_{13}\log of\;household\;asset_{i}\;+\;\beta_{14}social\_security_{i}+\beta_{15}gvt\_responsibility_{i}+\;\beta_{16}save\_retire_{i}+\beta_{17}save\_ltc_{i}+\beta_{18}parents\_inheritance_{i}+\;\beta_{19}parents\_educ_{i}+\beta_{20}one\_parentcare_{i}+\;\beta_{21}both\_parentcare_{i}+\varepsilon_{i}$$

Moreover, we conducted a subsample analysis by area (rural/urban) and gender, considering the regional gap.

## 3. Results

The full-sample regression results for perceptions of family caregiving norms are presented in Table 5. Starting with model 1, which includes basic demographic variables as control variables, certain variables were added in other models to understand the robustness of results and associations of the added variables. It is important to note that significant variables remain consistent in all models. The results show that the rural variable has a significantly positive association with family caregiving norms in all models. Moreover, household assets, government dependency, and when one parent requires care have significantly positive associations when social security has a significantly negative association with family caregiving norms. The regression results imply that perception towards family caregiving is still positively nurtured among respondents of rural areas more than their urban counterparts. A similar perception was also observed among respondents who have higher assets, are in favor of government support for long-term care, rely less on social security, and have one parent that requires care.

Since residency in rural and urban area is found to be an important consideration for forming perception towards family caregiving, we conducted a sub-sample analysis between residents of urban and rural areas. The results of the sub-sample analysis are presented in Table 6. Some variables that were not significantly associated with family caregiving attitude in full-sample analysis became significant in sub-sample analysis. The regression results show that females have a negative perception when the factors of being unemployed, having more household assets, having government support, and when one parent requires care have significantly positive associations with the traditional family caregiving norms. On the other hand, in urban areas, respondents who are married and dependent on government support have a significantly positive association when household assets and dependency on the social security have a significantly negative association with the traditional family caregiving norms.

To investigate the issue further, we have conducted sub-sample analysis between males and females of urban and rural areas. The results of the sub-sample analysis by region and gender are presented in Table 7. The results show that females in the rural areas who have more household assets, are dependent on government support, received family inheritance, and have one parent that requires care have positive associations with the traditional family caregiving norms. On the other hand, females in the urban areas, who are middle aged, and have = low education have negative associations when the factors of being unemployed, requiring government support for long-term care, and having one parent that requires care have positive associations with the traditional family caregiving norms. For rural males, positive perceptions are found among those who have higher education, are married, depend less on social security, are in favor of government support, have savings for long-term care, and did not receive family inheritance. Positive perception about traditional family caregiving is observed among rural males whose who have fewer household assets, are in favor of government support for long-term care, do not have savings for long-term care, and have parents who do not need care.

## 4. Discussion

The traditional family caregiving norms require family members, particularly female members, to take care for elderly family members [2,3,4,12]. However, changes in socio-economic conditions and demographic shifts are often found to impact the perception of traditional caregiving norms [1,14,15,16,17]. With this background, we investigated the association between demographic and socio-economic conditions and family caregiving norms in China. Moreover, we placed a special emphasis on family caregiving norms in rural and urban areas. In support of our hypothesis, the results of the study show that demographic and socio-economic factors have significant association with the traditional family caregiving norms. Respondents who have higher assets and do not rely much on social security are positive about family caregiving norms. This is inconsistent with the study of Fukuda et al. [1] and Khan et al. [38] that found no association of household assets and social security with family caregiving norms in Japan. Higher household assets and lower dependence on social security are indicators of economic solvency, too, which could reduce caregiving burden and stress and could also act as a deterrent for women to seek outside employment. Previous studies found that those in the lower-economic-capacity group could not afford the desired level of formal care and can therefore avoid providing informal family care [39,40,41,42]. However, respondents who are in favor of government support for the long-term care have a positive perception of the traditional family caregiving norms, which is consistent with previous studies [1,38]. This positive perception could emerge from the possibility that government support could be used for formal caregiving, which will complement the need for informal caregiving [43,44,45,46].

Regarding the rural–urban differences in familial caregiving, our study shows that people in rural areas have strong filial piety about caregiving. This is not the case with urban areas. This finding about the rural areas is consistent with that of Cong and Silverstein [47]. Furthermore, unlike filial traditions, rural women negatively perceive family caregiving norms, especially if they are employed and not wealthy. This implies that eldercare in rural areas may no longer rely on the traditional notion of female family members being the main family caregivers [48]. Similarly, middle-aged women of urban areas with higher education and who are employed are likely to have a negative perception towards traditional family caregiving. It seems that modernization has caused a reconstruction of the traditional perspective of filial piety [49,50].

These findings have several implications for policy. First, considering that government dependency leads to positive perceptions of family caregiving norms in rural areas, the government may support the valuable elderly in rural areas. This supports the argument of Li et al. [10] and Zhang [51], who claimed that rural residents have more difficulty accessing formal care than do urban residents. Second, the government may consider introducing a comprehensive long-term care system because traditional family caregivers negatively perceive family caregiving norms.

Nonetheless, this study had some limitations. First, the dataset we used does not completely represent China. As indicated in the Data section, the survey collected data from six major cities and the surrounding rural areas. Thus, there is room for improvement in terms of representativeness. Future studies may include more cities and towns with larger sample sizes. Thus, the representativeness of the results could be improved. Second, the dataset we used was prepared in 2013 and is relatively old. Hence, we may not have captured the latest trends in China. Third, there are no data to compare socio-economic changes in rural and urban areas to pinpoint why rural women have a negative perception compared to their urban counterparts. Finally, we have explained family caregiving norms using demographic and socio-economic aspects. There may be other issues that also influence perception of family caregiving norms, such as caregivers’ burden, stress, relationship with care recipients, and others. We were unable to use these variables due to the data limitations. Future studies should include all important aspects of family caregiving norms to provide a more comprehensive finding.

## 5. Conclusions

This study sheds light on the perceptions of family caregiving norms in China. To the best of our knowledge, this is the first quantitative study to investigate the perceptions of family caregiving norms in China, with a view comparable to that of neighboring Japan. This study quantitatively demonstrates how various demographic and socioeconomic conditions are associated with the perceptions of family caregiving norms in China. Finally, this study uniquely covered the aspect of rural–urban differences. The study revealed that rural residency, household assets, and government dependency positively impact the perception of care in China. A notable difference from the Japanese results was that Chinese women had a rather negative perception of family caregiving norms. Furthermore, urban–rural subsample analyses revealed that women in rural areas perceive caregiving negatively.

The findings of this study have implications for policymakers, government officials, and researchers. Family caregiving dynamics need to be studied and understood in a traditional-value-based country such as China due to ongoing changes in the socio-economic conditions of potential caregivers. The results clearly show the changing perception of family caregiving norms, particularly among rural women, suggesting a more negative perception of family caregiving. The government and other policymakers must respond to this transformation and provide more support to the elderly. Alternatively, the government could incorporate technologies and instruments supporting long-term care that are easily accessible so that the caregiving burden is eased.

## Figures and Tables

**Table 1:** Variable definitions.

Variables	Definition
Perception of family caregiving norms (dummy)	Child(ren) should take care of their parents when they require long-term care. (1 = completely disagree, 2 = somewhat disagree, 3 = can’t say which, 4 = somewhat agree, 5 = completely agree)
Rural (dummy)	1 = living in a rural area, 0 = living in an urban area
Female (dummy)	1 = female, 0 = male
Age	Age
Age_squared	Age squared
Years of education	Years of education
Unemployed (dummy)	1 = not employed, 0 = employed
Married (dummy)	1 = married, 0 = not married
Number of siblings	Number of siblings
Number of sons	Number of sons
Number of daughters	Number of daughters
Log of household income	Natural log of annual household income
Log of household asset	Natural log of household financial assets
Social security	The proportion of living expenses to cover using social security income after retirement
Government dependency (dummy)	It is the government’s responsibility to take care of the elderly financially. (1 = completely agree/agree, 0 = can’t say which/somewhat disagree/completely disagree)
Savings_retire (dummy)	1 = have retirement savings, 0 = no savings
Savings_ltc (dummy)	1 = have savings for long-term care, 0 = no savings
Inheritance from parents (dummy)	1 = have received an inheritance or monetary transfer of more than CNY 100,000 from their parents, 0 = no inheritance
Parents’ years of education	Average years of education of parents
Caring for one parent (dummy)	1 = either parent requires care, 0 = otherwise
Caring for both parents (dummy)	1 = both parents require care, 0 = otherwise
Cross_fem&age squared	An interaction term between women and age squared

**Table 2:** Descriptive statistics of the dependent and other control variables.

	Mean	Std. Dev.	Min	Max
Dependent Variable				
Perception of family caregiving norms	4.1079	0.7421	0	5
Control Variables				
Rural	0.3555556	0.478871	0	1
Female	0.497619	0.500193	0	1
Age	43.62222	13.6757	20	74
Age_squared	2089.775	1223.936	400	5476
Years of education	10.31429	3.398774	0	18
Unemployed	0.0222222	0.147464	0	1
Married	0.8095238	0.392833	0	1
Number of siblings	1.892063	1.584606	0	9
Number of sons	0.6174603	0.64981	0	4
Number of daughters	0.5087302	0.667469	0	4
Household income (CNY)	16,75,795	12,500,000	10,000	1 × 10^8^
Log of household income	11.27634	1.124813	9.21034	18.42068
Household assets (CNY)	8,093,490	27,100,000	0	1 × 10^8^
Log of household assets	10.16868	4.458747	0	18.42068
Social security	0.238373	0.242197	0	0.95
Government dependency	0.7738095	0.41853	0	1
Savings_retire	0.3571429	0.479348	0	1
Savings_ltc	0.2460317	0.430868	0	1
Inheritance from parents	0.0777778	0.267928	0	1
Parents’ years of education	6.50754	3.743692	0	16
Caring for one parent	0.2063492	0.404845	0	1
Caring for both parents	0.1095238	0.312419	0	1
Cross_fem&age squared	1064.404	1372.756	0	5329
Observations	1260			

**Table 3:** Perception of family caregiving norms by age group.

Perceptions of Family Caregiving Norms	Age				Total
	Less than or Equal to 40	41–50	51–60	Greater than 60	
1	1	0	0	1	2
	17.00%	0.00%	0.00%	52.00%	16.00%
2	12	6	2	3	23
	2.09%	2.44%	0.81%	1.55%	1.83%
3	101	31	42	31	205
4	17.63%	12.60%	16.94%	16.06%	16.27%
5	291	133	112	101	637
Total	50.79%	54.07%	45.16%	52.33%	50.56%
	168	76	92	57	393
F-statistics	F = 1.56				

**Table 4:** Perception of family caregiving norms by other demographic characteristics.

Perception of Family Caregiving Norm	Sex		Unemployed		Married		Area		Total
	Male	Female	Yes	No	Yes	No	Rural	Urban	
1	1	1	0	2	2	0	2	0	2
	16.00%	16.00%	0.00%	0.16%	0.20%	0.00%	0.45%	0.00%	0.16%
2	12	11	0	23	19	4	14	9	23
	1.90%	1.75%	0.00%	1.87%	1.86%	1.67%	3.13%	1.11%	1.83%
3	94	111	4	201	160	45	47	158	205
	14.85%	17.70%	14.29%	16.31%	15.69%	18.75%	10.49%	19.46%	16.27%
4	317	320	12	625	509	128	204	433	637
	50.08%	51.04%	42.86%	50.73%	49.90%	53.33%	45.54%	53.33%	50.56%
5	209	184	12	381	330	63	181	212	393
	33.02%	29.35%	42.86%	30.93%	32.35%	26.25%	40.40%	26.11%	31.19%
Total	633	627	28	1232	1020	240	448	812	1260
	100%	100%	100%	100%	100%	100%	100%	100%	100%
Mean difference	t = 1.4947		t = −1.2823		t = −1.5385		t = −4.1217 ***		

Note: The first and second lines of each row indicate the number and their percentages, respectively. The symbol *** indicates *p* < 0.01.

**Table 5:** Full-sample ordered probit regression results of perceptions of family caregiving norms with rural residency and other control variables.

Variables	Dependent Variable: Perception of Family Caregiving Norm				
	Model 1	Model 2	Model 3	Model 4	Model 5
Rural	0.3084 ***	0.2923 ***	0.3033 ***	0.2664 ***	0.2173 **
	(0.0825)	(0.0875)	(0.0874)	(0.0906)	(0.0955)
Female	−0.0410	−0.0464	−0.0417	−0.0518	−0.0561
	(0.1238)	(0.1237)	(0.1242)	(0.1268)	(0.1268)
Age	0.0049	0.0026	0.0036	0.0097	0.0051
	(0.0187)	(0.0197)	(0.0197)	(0.0199)	(0.0201)
Age_squared	−0.0000	0.0000	−0.0000	−0.0001	−0.0000
	(0.0002)	(0.0002)	(0.0002)	(0.0002)	(0.0002)
Education years	0.0043	0.0061	0.0059	0.0047	0.0096
	(0.0125)	(0.0125)	(0.0125)	(0.0127)	(0.0136)
Unemployed	0.3371	0.3389	0.3247	0.2976	0.3103
	(0.2158)	(0.2160)	(0.2188)	(0.2335)	(0.2351)
Married	0.0814	0.0596	0.0578	0.0919	0.0931
	(0.0936)	(0.0951)	(0.0960)	(0.0990)	(0.0988)
Cross_fem&age squared	−0.0000	−0.0000	−0.0000	−0.0000	−0.0000
	(0.0001)	(0.0001)	(0.0001)	(0.0001)	(0.0001)
Number of siblings		−0.0007	0.0007	−0.0110	−0.0116
		(0.0236)	(0.0236)	(0.0237)	(0.0237)
Number of sons		0.0434	0.0394	0.0410	0.0318
		(0.0612)	(0.0611)	(0.0615)	(0.0621)
Number of daughters		0.0557	0.0556	0.0410	0.0362
		(0.0575)	(0.0576)	(0.0579)	(0.0585)
Log of household income			−0.0150	−0.0131	−0.0109
			(0.0267)	(0.0266)	(0.0265)
Log of household asset			0.0148 *	0.0136 *	0.0127
			(0.0078)	(0.0077)	(0.0077)
Social security				−0.2531 *	−0.2490 *
				(0.1420)	(0.1427)
Government dependency				0.6801 ***	0.6802 ***
				(0.0800)	(0.0800)
Savings_retire				0.0190	0.0199
				(0.0834)	(0.0834)
Savings_ltc				0.0481	0.0319
				(0.0893)	(0.0899)
Parents inheritance					−0.1349
					(0.1169)
Parents education years					−0.0110
					(0.0122)
One parent care					0.1936 *
					(0.1152)
Both parent care					−0.1093
					(0.1510)
Observations	1260	1260	1260	1260	1260
Adj R-squared	0.0103	0.0107	0.0122	0.0446	0.0464
Log likelihood	−1355	−1355	−1353	−1309	−1306
Chi2 statistics	26.11	28.12	35.23	125.1	131.6
*p*-value	0.00	0.00	0.00	0.00	0.00

Robust standard errors in parentheses. *** *p* < 0.01; ** *p* < 0.05; * *p* < 0.1.

**Table 6:** Subsample ordered probit regression results of perceptions of family caregiving norms by rural/urban residency.

Variables	Dependent Variable: Perception of Family Caregiving Norm	
	Rural	Urban
Female	−0.2836 **	−0.0768
	(0.1160)	(0.0802)
Age	−0.0493	0.0229
	(0.0353)	(0.0255)
Age_squared	0.0006	−0.0002
	(0.0004)	(0.0003)
Education years	0.0014	0.0221
	(0.0215)	(0.0179)
Unemployed	0.8345 *	0.1673
	(0.4690)	(0.2754)
Married	−0.0247	0.2246 *
	(0.1907)	(0.1226)
Number of siblings	0.0028	−0.0324
	(0.0353)	(0.0329)
Number of sons	0.0350	−0.0177
	(0.0960)	(0.0844)
Number of daughters	0.1037	−0.0678
	(0.0903)	(0.0773)
Log of household income	−0.0230	−0.0086
	(0.0288)	(0.0790)
Log of household asset	0.0229 **	−0.0214 *
	(0.0095)	(0.0123)
Social security	−0.0262	−0.3040 *
	(0.2759)	(0.1682)
Government dependency	1.1986 ***	0.4534 ***
	(0.1470)	(0.0932)
Savings_retire	0.0010	0.0220
	(0.1501)	(0.1051)
Savings_ltc	−0.0575	0.0671
	(0.1830)	(0.1062)
Parents inheritance	−0.5302	−0.0980
	(0.4399)	(0.1239)
Parents education years	−0.0116	−0.0162
	(0.0207)	(0.0154)
One parent care	0.5169 ***	0.0102
	(0.1866)	(0.1522)
Both parent care	−0.1918	−0.0852
	(0.2131)	(0.2279)
Observations	448	812
Adj R-squared	0.109	0.0259
Log likelihood	−436.5	−833.9
Chi2 statistics	104.4	45.50
*p*-value	0.00	0.00

Robust standard errors in parentheses. *** *p* < 0.01; ** *p* < 0.05; * *p* < 0.1.

**Table 7:** Subsample ordered probit regression results of perceptions of family caregiving norms by rural/urban residency and gender.

Variables	Dependent Variable: Perception of Family Caregiving Norm			
	Rural	Urban	Rural	Urban
	Female		Male	
Age	0.0093	−0.1440 ***	0.0220	0.0348
	(0.0491)	(0.0498)	(0.0348)	(0.0382)
Age_squared	−0.0001	0.0018 ***	−0.0003	−0.0003
	(0.0005)	(0.0006)	(0.0004)	(0.0004)
Education years	0.0480	−0.0567 *	0.0452 *	−0.0081
	(0.0301)	(0.0328)	(0.0268)	(0.0246)
Unemployed	0.7094	4.6527 ***	0.2120	0.2876
	(0.6286)	(0.2899)	(0.3010)	(0.4318)
Married	−0.3472	0.1384	0.3276 **	0.1873
	(0.3448)	(0.2611)	(0.1631)	(0.1877)
Number of siblings	0.0187	−0.0500	−0.0378	−0.0345
	(0.0532)	(0.0500)	(0.0448)	(0.0507)
Number of sons	0.1515	0.0208	−0.0144	−0.1033
	(0.1419)	(0.1407)	(0.1203)	(0.1277)
Number of daughters	0.0870	0.1003	0.0169	−0.2057
	(0.1263)	(0.1402)	(0.0977)	(0.1333)
Log of household income	−0.0088	−0.0169	−0.1429	0.0772
	(0.0515)	(0.0393)	(0.1058)	(0.1219)
Log of household asset	0.0294 **	0.0210	−0.0025	−0.0403 **
	(0.0144)	(0.0135)	(0.0170)	(0.0183)
Social security	0.0230	0.0863	−0.4827 *	−0.1275
	(0.4118)	(0.4160)	(0.2515)	(0.2268)
Government dependency	1.1789 ***	1.3444 ***	0.4330 ***	0.4708 ***
	(0.2466)	(0.2002)	(0.1369)	(0.1284)
Savings_retire	0.0977	−0.0711	0.0609	−0.0150
	(0.2006)	(0.2293)	(0.1483)	(0.1523)
Savings_ltc	−0.1846	−0.0142	0.3258 **	−0.2602 *
	(0.2330)	(0.2984)	(0.1494)	(0.1534)
Parents inheritance	4.0923 ***	−0.5355	−0.2867 *	0.0275
	(0.3752)	(0.4343)	(0.1578)	(0.1923)
Parents education years	−0.0262	0.0058	−0.0201	−0.0131
	(0.0287)	(0.0318)	(0.0230)	(0.0212)
One parent care	0.6797 **	0.4598 *	−0.0837	0.1045
	(0.2895)	(0.2487)	(0.2210)	(0.2174)
Both parent care	−0.6800 **	0.3977	0.3466	−0.6195 **
	(0.3269)	(0.3187)	(0.3408)	(0.3032)
Observations	225	223	402	410
Adj R-squared	0.114	0.163	0.0457	0.0373
Log likelihood	−219.5	−200.1	−408.3	−411.3
Chi2 statistics	451.1	543.6	38.05	36
*p*-value	0	0	0.00381	0.00706

Robust standard errors in parentheses. *** *p* < 0.01; ** *p* < 0.05; * *p* < 0.1.

## Data Availability

Data are available upon request.
